# Regulation of the cell cycle and PI3K/Akt/mTOR signaling pathway by tanshinone I in human breast cancer cell lines

**DOI:** 10.3892/mmr.2021.12080

**Published:** 2021-04-12

**Authors:** Li Wang, Jianzhong Wu, Jianwei Lu, Rong Ma, Dawei Sun, Jinhai Tang

Mol Med Rep 11: 931-939, 2015; DOI: 10.3892/mmr.2014.2819

Following the publication of the above paper, a concerned reader drew to the Editor's attention that Fig. 5 contained apparent anomalies, including unexpectedly similar-looking cells and repeated patternings of the cells in terms of their layout/arrangement within the data panels.

After having conducted an independent investigation in the Editorial Office, the Editor of *Molecular Medicine Reports* has determined that the above paper should be retracted from the Journal on account of a lack of confidence regarding the authenticity of the data. The authors were asked for an explanation to account for these concerns, but the Editorial Office never received any reply. The Editor regrets any inconvenience that has been caused to the readership of the Journal.

